# An Unusual Case of Thyroid Storm Masquerading as an Intestinal Obstruction in a Patient With Malrotation of the Gut

**DOI:** 10.7759/cureus.13948

**Published:** 2021-03-17

**Authors:** Khalid Bashir, Zeenat K Bakhsh, Hesham A Gad, Mohammed T Bashir, Amr Elmoheen

**Affiliations:** 1 Emergency Medicine, Hamad Medical Corporation, Doha, QAT; 2 College of Medicine, Qatar University, Doha, QAT; 3 Emergency Medicine, Weill Cornell Medical College, Doha, QAT; 4 Endocrinology and Diabetes, Arab Contractors Hospital, Cairo, EGY; 5 School of Medicine and Dentistry, University of Aberdeen, Scotland, GBR

**Keywords:** thyrotoxicosis, small bowel obstruction, malrotation, emergency medicine

## Abstract

Gut malrotation is usually a pediatric condition that presents mainly in the first month of life. It rarely presents in adults and is often a significant diagnostic dilemma to the surgeon. Failure to diagnose and treat this condition early may result in dire consequences like small gut gangrene. A previously healthy, 41-year-old female patient presented to the emergency department with a three-day history of lower abdominal pain and constipation. On examination, she had tachycardia, and her abdominal examination was soft, with mild tenderness in the hypogastric region and scanty bowel sounds. Urine pregnancy and dipstick were unremarkable. The patient was diagnosed with small bowel obstruction (malrotation of the small bowel loops with twisting of the mesenteric vessels). The patient was found to have thyrotoxicosis, which was the cause of the intestinal obstruction. She received conservative treatment and specific thyrotoxicosis management (carbimazole, cholestyramine, Lugol’s iodine, and intravenous hydrocortisone). The symptoms resolved entirely after a two-day stay at the intensive care unit.

Small bowel malrotation is a congenital anomaly and can present with abdominal pain and obstruction in adulthood. Thyrotoxicosis can cause small bowel obstruction if there is underlying malrotation.

## Introduction

Intestinal malrotation is a clinical condition characterized by a partial or complete failure of the midgut’s counterclockwise rotation around the fetal mesenteric vessels [1]. It is a rare condition that occurs in one in every 200-500 newborns [2]. Symptomatic cases have a prevalence of one in 6000 newborns [2]. This condition rarely presents in adults. A diagnosis of intestinal malrotation in adults requires a high index of suspicion.

Thyrotoxicosis, on the other hand, is a rare diagnosis in the emergency department [3] and thyroid storm has a high mortality rate range from 8%-25% [4]. Uncommon symptoms of thyrotoxicosis include abdominal pain and vomiting. Associating these uncommon symptoms with transaminitis and other laboratory abnormalities may result in an error in diagnosis, usually hepatitis [1]. Thyrotoxicosis usually affects several cellular metabolic processes. The excess production of thyroid hormone results in heat intolerance, tachycardia, palpitations, fatigue, tremulousness, and sweating [1]. Because thyrotoxicosis may be life-threatening if not treated on time, early diagnosis via thyroid function tests and intervention with beta-blockers may be necessary to minimize thyroid hormone production and secretion.

We present a case of small bowel obstruction due to malrotation that presented with thyrotoxicosis.

## Case presentation

A previously healthy, 41-year-old patient presented to the emergency department with a three-day history of lower abdominal pain and constipation. She never smoked or consumed alcohol. On examination, her blood pressure was 134/69 mmHg, and her pulse was 133 beats/minute. Abdominal examination showed tenderness in the hypogastric region and scanty bowel sounds. Urine pregnancy and dipstick were unremarkable.

Her complete blood count showed white blood cells (WBCs) of 6.5 x10^3/uL, hemoglobin of 8.6 gm/dL, and platelets of 331 x10^3/uL. Her renal function showed urea of 5.0 mmol/L and creatinine of 49 umol/L. Her beta-human chorionic gonadotropin (BHCG) was <1 mIU/mL.

The thyroid function test showed a low thyroid stimulating hormone (TSH) <0.01 miu/L (0.3-4.20 miu/L), a high thyroxine (T4) 54.5 pmol/L (11-23.3 pmol/L), a high triiodothyronine (T3) 8.8 pmol/L (3.7-8.4 pmol/L), and TSH receptor antibody 7.7 iu/L (n<1.75 iu/L).

**Figure 1:**
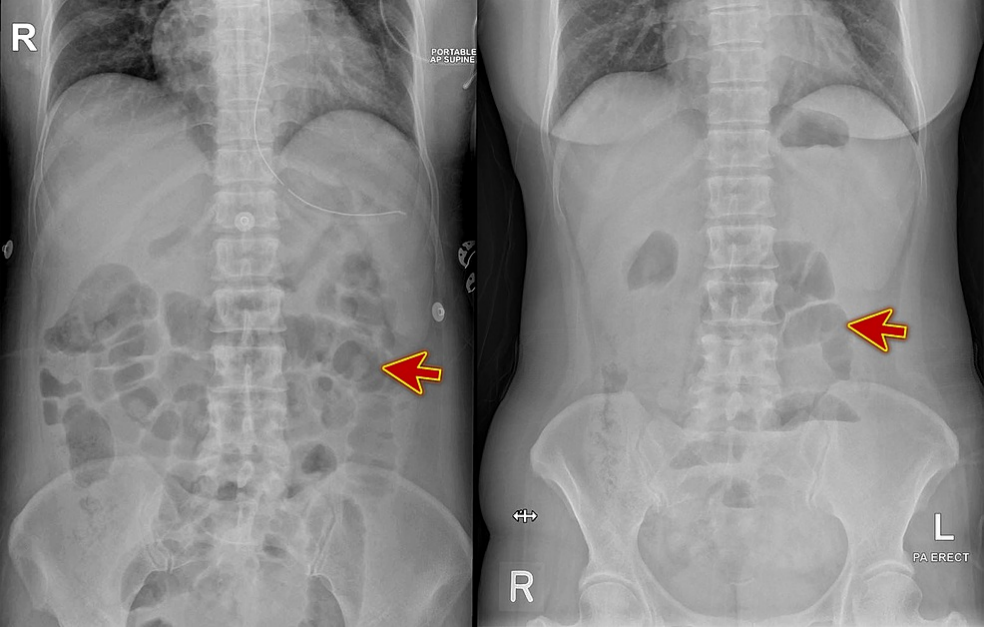
The X-rays of the abdomen revealed dilated small bowel loops (red arrows) with multiple fluid levels

The X-rays of the abdomen at initial presentation and six hours later revealed dilated small bowel loops with multiple fluid levels (Figure 1).

**Figure 2:**
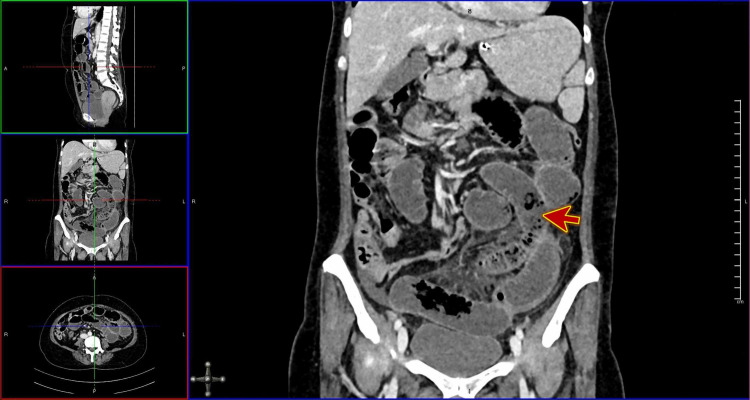
Computed tomography (CT) scan with contrast of the abdomen showing small bowel obstruction (red arrow)

**Figure 3:**
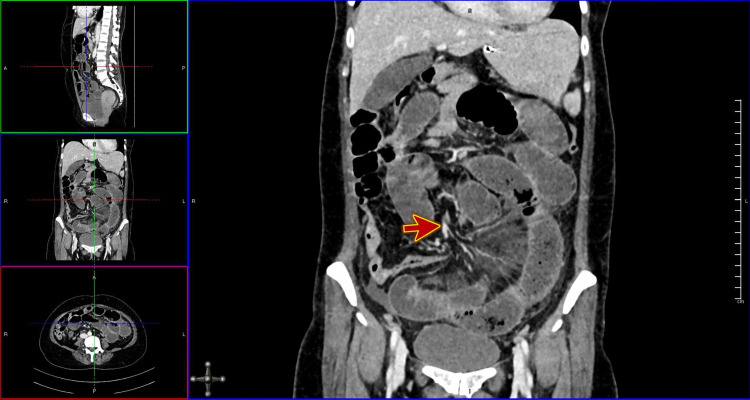
Computed tomography (CT) scan with contrast of the abdomen showing malrotation of the small bowel loops with twisting of the mesenteric vessels (red arrow)

Computed tomography (CT) scan with contrast confirmed a small bowel obstruction; a small bowel diameter of 34 mm; and malrotation of the small bowel loops with twisting of the mesenteric vessels (Figures 2-3).

She was started on conservative treatment with intravenous fluids infusion, nil orally, placement of a nasogastric tube, which produced yellowish output, and specific thyrotoxicosis management such as carbimazole 20 mg every eight hours, cholestyramine, Lugol’s iodine, and intravenous hydrocortisone.

She stayed in the intensive care unit for two days, and her symptoms resolved completely with the conservative treatment. She was able to tolerate oral intake and open bowels normally, and her heart rate came back to normal.

The patient was discharged to follow up at the surgical outpatient department to operate on her on an elective basis to prevent a future episode of intestinal obstruction or strangulation.

## Discussion

Like the patient in our study, the risk for thyrotoxicosis is usually high for women in their fourth through sixth decades of life [5]. While the patient in this case study was not pregnant, it is important to note that exposure to high levels of thyroid hormone in utero contributes to high rates of spontaneous abortion. Studies have documented rates as high as 22% [6].

Thyrotoxicosis also presents as diabetic ketoacidosis, hypokalemic periodic paralysis, and cardiac arrhythmias [7-8]. A case reported in 2000 revealed multiorgan dysfunction associated with liver failure and lactic acidosis [9].

Because most emergency departments do not offer rapid thyroid function tests, the diagnosis of thyrotoxicosis is mainly clinical. Hyperreflexia, diffuse goiter, and persistent tachycardia are major clinical symptoms that could point to thyrotoxicosis. Other factors that may be considered diagnostic are high thyroxine (T4) and triiodothyronine (T3) levels with low thyroid-stimulating hormone [5]. Modest glucose elevations (usually in 30% of patients), transaminases and bilirubin, and calcium (in 10% of cases) may present, alongside a mild normochromic normocytic anemia [5].

Malrotation in adults is an uncommon occurrence, usually resulting in multiple visits to the hospital and leaving an inexperienced surgeon in a state of confusion [10-11]. Malrotation may also present acutely, owing to midgut volvulus, leading to the intestines’ bowel gangrene and ischemia. Of course, the outcome is usually fatal and results in death or short bowel syndrome if the patient survives. The key to survival is early diagnosis and treatment [12]. A high clinical suspicion index is necessary, mostly in cases with recurrent episodes of bilious vomiting and abdominal pain, without a history of tuberculosis or surgery. It is also important to note that plain radiographs are of very little use in diagnosis. Contrast-enhanced CT scan remains the choice investigation in adults [12]. Investigations usually reveal a reversed relation of the superior mesenteric vein and superior mesenteric artery, upper right abdominal bowel loops, inability to visualize the caecum, righted duodenojejunal flexure, and dilatation of duodenal loops.

There are several types of gut malrotation described in the literature such as 1 to 3 with their subtypes [13]. The exact frequency of each type is not well-known to cause a lot of patients to remain asymptomatic and are diagnosed on CT scans or during surgery. In our case, we are not sure of the exact type of malrotation, as the patient is currently awaiting surgical consultation and was managed conservatively.

Thyroid hormone is a general metabolic hormone, which plays an important role in gut motility, water, and electrolyte balance in addition to other important functions [4]. One plausible explanation in our case was that an abnormally high level of thyroxine resulted in increased motility of the gut and led to subacute intestinal obstruction due to pre-existing congenital gut malrotation. Similarly, a case was reported where thyroid storm resulted in intestinal obstruction and bowel ischemia required surgery and partial resection of the intestine [14].

Clinical management of thyrotoxicosis is dependent on clinical presentation. With careful outpatient care, the clinician can manage mild hypothyroidism. On the other hand, moderate to severe thyrotoxicosis calls for emergency treatment. Firstline therapy revolves around stabilization: oxygenation, airway protection, cardiorespiratory monitoring, and intravenous fluid monitoring [15]. Acetaminophen may be used to manage fever. In the event of dehydration, fluid replacement may be employed. The clinician may also start beta-blockers and anti-thyroid medications while precipitating trauma, infection, myocardial infarction, cerebrovascular accident, and medication reactions, and managing them accordingly [15].

While our case may be an unusual presentation of a rare medical condition, it is important to emphasize the learning points from the first visit to the emergency department. Our patient presented with tachycardia as evidenced by a pulse rate of 133 beats per minute, lower abdominal pain, and constipation. Persistent tachycardia indicates that a search is done for other potential causes like pulmonary embolism, acute anemia, sepsis, arrhythmia, hypnotic/sedative withdrawal, ingestion of anticholinergic or sympathomimetic agents.

After successful stabilization, the clinician should aim to inhibit the synthesis and release of hormones, prevent T4 conversion to T3, and impede the action of hormones in the periphery [5]. Thioamides (methimazole and propylthiouracil) may be used for the inhibition of synthesis, as they block thyroid peroxidase. Our patient was treated with IV fluids, nil orally, and specific thyrotoxicosis management, such as carbimazole, cholestyramine, Lugol’s iodine, and intravenous hydrocortisone, and showed drastic improvement within two days of stay in the intensive care unit (ICU).

## Conclusions

Thyrotoxicosis can present as acute abdomen, and early diagnosis is important. Failure to treat this condition on time may result in a thyroid storm, which has a high mortality rate. Unexplained tachycardia must be evaluated. Thyrotoxicosis may present as intestinal obstruction, especially in a patient with underlying gut malrotation.
